# Multimodal conservative management of arthrofibrosis after total knee arthroplasty compared to manipulation under anesthesia: a feasibility study with retrospective cohort comparison

**DOI:** 10.1186/s40814-022-01026-y

**Published:** 2022-03-25

**Authors:** Michelle R. Rauzi, Jared R. H. Foran, Michael J. Bade

**Affiliations:** 1 Physical Therapy Program, Department of Physical Medicine and Rehabilitation, University of Colorado, 13121 E 17th Ave, Aurora, CO 80045 USA; 2 Panorama Orthopedics & Spine Center, 660 Golden Ridge Rd. #250, Golden, CO 80401 USA; 3 Veterans Affairs Geriatric Research, Education and Clinical Center, VA Eastern Colorado Healthcare System, 13611 East Colfax, Aurora, CO 80045 USA

**Keywords:** Static progressive splinting, Manual therapy, Physical therapy, Rehabilitation, Arthrofibrosis, Total knee arthroplasty

## Abstract

**Background:**

The ideal treatment of early-stage arthrofibrosis after total knee arthroplasty is unclear. The purpose of this study was to determine the treatment effect, including variability, and feasibility of a multimodal physical therapy program as compared to manipulation under anesthesia.

**Methods:**

This was a prospective feasibility study with a retrospective cohort comparison. Ten consecutive patients (aged 64 ± 9 years, 7 females) with early-stage arthrofibrosis were enrolled 6 weeks after primary total knee arthroplasty and participated in the multimodal physical therapy program. The multimodal physical therapy program consisted of manual therapy, therapeutic exercise, and static progressive splinting delivered over 4 weeks. The outcomes included knee range of motion (ROM), adherence, patient satisfaction, and safety. Data were compared to a retrospective cohort of 31 patients with arthrofibrosis (aged 65 ± 9 years, 20 females) who underwent manipulation under anesthesia followed by physical therapy.

**Results:**

Overall, knee ROM outcomes were similar between multimodal physical therapy (110° ± 14) and manipulation under anesthesia (109° ± 11). Seven out of ten patients achieved functional ROM (≥ 110°) and avoided manipulation under anesthesia with the multimodal physical therapy program. Three out of 10 multimodal physical therapy patients required manipulation under anesthesia secondary to failure to demonstrate progress within 4 weeks of the multimodal physical therapy program. Adherence to the multimodal physical therapy program was 87 ± 9%. The median patient satisfaction with the multimodal physical therapy program was “very satisfied.” Safety concerns were minimal.

**Conclusion:**

The use of the multimodal physical therapy program is feasible for treating early-stage arthrofibrosis after total knee arthroplasty, with 70% of patients avoiding manipulation under anesthesia. Randomized controlled trials are needed to determine the efficacy of the multimodal physical therapy program and to determine the optimal patient selection for the multimodal physical therapy program versus manipulation under anesthesia.

**Trial registration:**

ClinicalTrials.gov, NCT04837872.

**Supplementary Information:**

The online version contains supplementary material available at 10.1186/s40814-022-01026-y.

## Key messages regarding feasibility

- What uncertainties existed regarding the feasibility? The uncertainties regarding feasibility for the trial were the rate of recruitment, adherence and satisfaction of the static progressive splint, and preliminary responsiveness to the multimodal physical therapy intervention.
- What are the key feasibility findings? The key feasibility findings were as follows: (1) recruitment of 10 participants took 9 months from one surgeon, (2) participants were adherent to the splint protocol (86.6 ± 9.0%), (3) participants were satisfied with the splint (median 6 “very satisfied”), and (4) 7 out of 10 participants avoided manipulation under anesthesia showing favorable response to the multimodal physical therapy intervention for knee range of motion.
- What are the implications of the feasibility findings for the design of the main study? The implications for the design of the future efficacy study are that to recruit the number of needed participants, it will be necessary to conduct a multi-site trial. This has further implications for standardization of the manipulation under anesthesia procedure across surgeons/facilities and intervention fidelity across physical therapy clinics.

## Background

Total knee arthroplasty (TKA) remains the primary intervention for individuals with end-stage knee osteoarthritis (OA) who have not responded to conservative therapies. However, outcomes following TKA are variable with up to 20% reporting dissatisfaction [1]. The reasons for patient dissatisfaction are knee stiffness and persistent pain. The incidence of knee stiffness, also termed arthrofibrosis, ranges widely from 1.3 to 13% depending on the authors’ definition and diagnostic criteria [2–6]. Recently, an international panel of medical experts developed a consensus definition for knee arthrofibrosis and defined it as “restricted range of motion (ROM), in flexion or extension, that is not attributable to an osseous or prosthetic block to movement from mal-positioned or incorrectly sized components, metal hardware, ligament reconstruction, infection (septic arthritis), pain (complex regional pain syndrome), or other specific causes and is due to soft tissue fibrosis that was not present preoperatively” [4]. It may be further described by the time of development, early stage (< 3–6 months postoperative) versus late stage (> 6 months postoperative), and by severity (mild, moderate, severe) based on the following ROM restrictions respectively: flexion range of 90 to 100°, 70 to 89°, or < 70° or extension deficit of 5 to 10°, 11 to 20°, or > 20° [4]. Multiple risk factors are believed to contribute to postoperative arthrofibrosis including preoperative (i.e., high body mass index, female gender, limited ROM, and/or prior knee surgery), peri-operative (i.e., inappropriate prosthesis selection and/or alignment), and postoperative (i.e., inappropriate physical therapy, inadequate pain control, and/or dysregulation of inflammation) [7–10].

There is no clear effective treatment for arthrofibrosis following TKA. The first-line treatment option for early-stage arthrofibrosis is typically manipulation under anesthesia (MUA) since it is a non-invasive alternative to surgical options. However, there is disagreement surrounding the MUA procedure related to the techniques employed, appropriate timing following index TKA, and appropriateness for addressing knee extension deficits. In general, there is a consensus MUA should happen within 3 months of index TKA [11, 12]. Some authors argue for even earlier intervention, performing MUA as early as 4 weeks after index TKA [13] while others report similar gains of knee ROM for late (i.e., > 3 months) MUA [14–16]. Manipulation under anesthesia is also associated with an increased risk of adverse events including hemarthrosis, supracondylar fracture, and extensor mechanism disruption [6, 8, 9, 17]. The outcomes following MUA are variable, and 4 to 26% of individuals experience poor outcomes and continued knee stiffness [6, 9, 15–17]. The wide range of failure rates is related to varying definitions of failure though most authors consider final knee flexion ROM < 90° to be the threshold for an unsatisfactory outcome [12, 16]. If the initial treatment with MUA is not successful, failure rates increase and are approximately 50% following a second MUA [12, 18]. Furthermore, patients experience increased pain, swelling, reduced ROM, and impaired physical function immediately following MUA. Therefore, there is a need to identify other potential treatments to improve outcomes and mitigate risks associated with MUA for individuals presenting with arthrofibrosis in the early postoperative phase (< 3–6 months).

Physical therapy approaches including manual therapy, therapeutic exercise, and splinting are a potential alternative conservative option to MUA. The use of these interventions by physical therapists is variable due to the lack of consensus and lack of published protocols to guide clinical decision-making. Manual therapy has been shown to be effective as part of a multimodal program to improve pain, stiffness, and function in individuals with knee OA [19]; however, utilization of these techniques has not been studied after TKA. Therapeutic exercise has been studied more in this population, resulting in a stronger evidence base supporting the use of exercise following TKA; however, there is a lack of published protocols containing sufficient detail to reproduce them clinically, particularly, in regard to the patient with arthrofibrosis [20, 21]. As a result, there continues to be variability in practice especially with the prescription of ROM exercises. Thus, there is a need to establish manual therapy and therapeutic exercise protocols to manage individuals with early-stage arthrofibrosis. Additionally, evidence specific to static progressive splinting for arthrofibrosis after TKA has been limited to the use after the early postoperative phase and after failure to respond to MUA [22–24]. There have been no studies examining the effectiveness of using static progressive splinting in the early postoperative phase (i.e., within 3 months) nor before performing MUA. Furthermore, there have been no studies employing a structured approach using manual therapy, therapeutic exercise, and static progressive splinting prior to MUA.

Therefore, the purpose of this study was to determine the treatment effect, including variability, and feasibility of a multimodal physical therapy (MPT) program including manual therapy, therapeutic exercise, and static progressive splinting in individuals presenting with early-stage arthrofibrosis (6 weeks after index TKA) as this time period has been associated with improved outcomes following MUA. The results from this feasibility study were compared to a retrospective cohort who underwent MUA followed by physical therapy.

## Methods

### Study design and patients

This was a feasibility study with a retrospective cohort comparison. Subjects were consecutively recruited (*N* = 10) from a single orthopedic surgeon’s office (JF) from May, 2017, to January, 2018, and enrolled into the MPT group. Subjects were included if they had undergone a unilateral, primary TKA for end-stage OA and developed knee stiffness in the first 6 weeks after surgery as defined by knee flexion ROM of less than 100°. Subjects were also included if they presented with knee extension deficit, but the primary inclusion criteria were based on knee flexion deficit. Subjects were excluded if preoperative ROM was less than 15–110°; intraoperative (after capsular closure) ROM was less than 0–120°; if they had radiographic signs of heterotopic ossification, malaligned components, or component-related failures that could be responsible for difficulties with motion; or signs and symptoms consistent with joint infection or complex regional pain syndrome. Written informed consent was obtained from all participants. Subjects in the MPT group were compared to a retrospective cohort of individuals (*N* = 31) who met the same inclusion and exclusion criteria as the MPT group and were treated with MUA by the same surgeon (JF) between the dates of June, 2012, and February, 2016. Nineteen individuals who received MUA during this time period were excluded for the following reasons (*N*): preoperative ROM less than 15–110° (11), missing data (6), and pre-MUA ROM greater than 100° (2). This study was approved by the Catholic Health Initiatives Institute for Research and Innovation Institutional Review Board.

### Interventions

#### Index TKA and MUA

All surgeries were performed by the same orthopedic surgeon (JF). All implants utilized were cruciate-retaining, and surgeries were performed via a mid-vastus arthrotomy. The patella was resurfaced in all cases. Pain management protocol was as follows during the study period: patients were given a preoperative dose of 1000 mg Tylenol. Intraoperatively, they were given spinal anesthesia. For patients under the age of 70, postoperatively, they were given oxycodone 5–10 mg every 4 h as needed, meloxicam 15 mg once daily for 4 weeks, and Lyrica 75 mg twice daily for 2 weeks. If pain was uncontrolled with oxycodone, patients were given MS Contin or Oxycontin. For severe uncontrolled pain postoperatively, patients were given a single femoral nerve or adductor canal block (which lasted for approximately 12 h). Patients in both groups were prescribed physical therapy two to three times per week following surgery and evaluated clinically at 4–6 weeks after TKA for arthrofibrosis.

For patients in the MUA group, all MUA procedures were performed by the same board-certified orthopedic surgeon (JF). Each MUA was done using a standard protocol. Patients were administered propofol anesthesia in the operating room. They were considered properly sedated when they no longer resisted knee movement or adversely responded to painful stimuli. First, extension deficits were addressed by exerting downward pressure directly on the knee; care was taken to avoid excessive force into the extension to reduce the risk for complications. Next, the hip was flexed, and the knee was firmly yet carefully flexed until audible and palpable scar tissue was released. Following the manipulation, the knee was injected with an admixture of 1 cc of DepoMedrol 40 and 9 cc of 0.5% Marcaine under sterile conditions. The knee was then wrapped with an ACE bandage to discourage hemarthrosis. All patients were discharged to home shortly after the procedure. Patients were prescribed physical therapy twice per week for 8 weeks following MUA.

#### MPT program

The MPT program was conducted at two participating outpatient physical therapy clinics. All patients were seen twice per week for 4 weeks by licensed physical therapists trained in the MPT program. The MPT program consisted of manual therapy, therapeutic exercise, and utilization of a static progressive splint (Joint Active Systems SPS Knee, Effingham, IL). Although the primary focus of this study was on improving knee flexion ROM, two protocols were developed: one to improve flexion deficits and one to improve extension deficits. Full details of each protocol are reported in Additional files 1 and 2.

The major goal of the MPT program was to improve functional mobility by focusing on improved soft tissue and joint mobility and reduced pain. Techniques that were overly aggressive and led to increases in pain were not utilized as this may lead to an increase in the fibrotic/inflammatory response and negatively impact the patient’s willingness to move their knee during or after therapy [7]. Core manual therapy techniques were utilized at the initial evaluation to determine patient response and optimal technique selection. Techniques that facilitated improved within-session gains of ROM and/or reductions of pain were utilized throughout the MPT program. Manual therapy techniques that were easy to apply by the patient (e.g., patellofemoral mobilizations or soft tissue mobilization) were also prescribed as part of a home exercise program to be performed daily as appropriate. All patients were prescribed an active-assisted ROM exercise to be performed at least five times per day (recommended hourly while awake) and instructed to complete this exercise to their current limit of flexion and extension in a non-pain increasing manner. Patients were also prescribed flexibility exercises to be performed three times daily for 60 s each. Finally, patients were prescribed weight-bearing and task-specific exercises to be performed daily to incorporate ROM gains and facilitate strength gains within newly acquired ranges.

A static progressive splint was custom fit to each patient at initial evaluation. Patients were instructed to gradually increase the use of the splint to three times per day, 30 min a session, for a total of 90 min per day. Patients were instructed to increase the stretch delivered by the splint to a level of 2–3 (light stretch) out of 10 where 0 equaled “no stretch” and 10 equaled “painful stretch.” Every 5 min, patients were instructed to evaluate the level of stretch and increase or decrease the splint tension to maintain a level of 2–3 throughout the entire session. Patients with an extension deficit of > 5° were also instructed to utilize the splint to improve knee extension three times per day, 30 min a session, for a total of 90 min per day. Patients with 5° of knee extension deficit or less were instructed to stretch their knee in a gravity-assisted position that achieved a level of 2–3 stretch intensity for 30 min daily.

Patients were reassessed at 4 weeks (T1) to determine response to the MPT protocol and identify if functional ROM (≥ 110°) was achieved (see also Additional file 1). Functional ROM was defined as ≥ 110° of knee flexion. Although 90° is commonly used as a cutoff for functional knee flexion ROM, 110° was chosen because some activities of daily living require > 90° of knee flexion including tying shoes (105°) and lifting objects from the floor via squatting (116°) [25]. Also, some leisure activities, such as cycling, require knee flexion ROM up to 109° [26]. Individuals who were assigned to the MPT group but failed to achieve functional ROM ≥ 110° of knee flexion after 4 weeks (T1) were offered the option of MUA.

### Outcomes

**Fig. 1:**
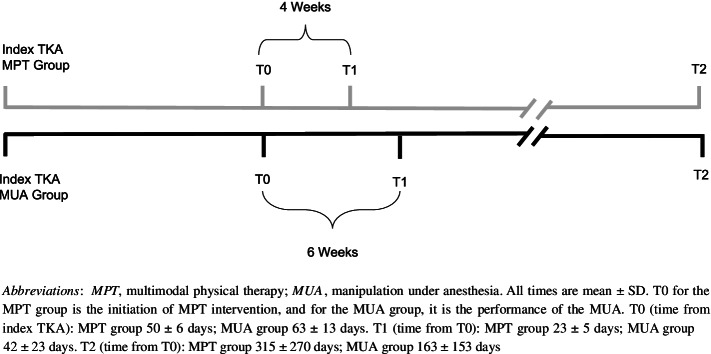
Timeline of outcome assessments

In the MPT group, outcome measures were assessed preoperatively, at referral to MPT (T0), at 4 weeks post-intervention (T1), and at the latest postoperative follow-up (T2—mean 315 days). In the MUA group, the outcome measures were obtained preoperatively, at the time of MUA (T0), at 6 weeks after MUA (T1), and at the latest postoperative follow-up (T2—mean 163 days). The timeline of assessments is reported in Fig. 1.

#### ROM

Passive knee flexion and extension ROM achieved at T1 was the main time point of interest. ROM was measured by standard goniometry as described by Norkin and White [27]. ROM was also assessed at T2. All ROM measurements were performed by the same individual (JF) who was not blinded.

#### Feasibility

Feasibility outcomes included recruitment, adherence to splint use, satisfaction of splint, and safety. Recruitment was measured by time (months) to recruitment completion, percent of total primary TKA procedures performed, and number of patients who declined enrollment. Adherence to splint use was tracked with a log completed by participants; they were instructed to place a checkmark for each day they used the JAS splint per protocol (30-min sessions, 3 times daily). Adherence was calculated as days of splint use completed divided by days of splint use prescribed; a priori adherence levels were considered acceptable if the mean was ≥ 80%. Satisfaction was assessed with a 7-point Likert scale ranging from 1 “extremely dissatisfied” to 7 “extremely satisfied.” Participants responded based on the following instruction: *please rate your satisfaction with your knee splint on the scale*. A priori satisfaction was considered acceptable if the median score was ≥ 4 (“somewhat satisfied” or higher). Adherence and satisfaction were assessed at T1 by treating therapists who were not blinded. Safety was assessed and tracked during each therapy session and physician follow-up; all adverse events were recorded.

### Data analysis

Descriptive statistics were calculated for baseline demographic variables, ROM outcomes over time, and feasibility outcomes. All data are reported as mean ± standard deviation. For knee extension, positive values indicate an extension deficit (lag) and negative values represent hyperextension. Only mean group differences and 95% confidence intervals (CI) are reported and were generated using independent *t*-tests; *p*-values are not reported since this feasibility study was not powered to detect differences. The mean differences and variability estimates align with recommended reporting standards for pilot and feasibility studies [28, 29]. Data from all participants in MPT are reported at T1 (*n* = 10) as well as data from individuals who met the goal ≥ 110° of knee flexion by T1 (*n* = 7). At T2, only data from individuals who met the goal ≥ 110° of knee flexion at T1 are reported, and the three individuals who underwent MUA following the MPT program are reported separately.

## Results

During the study period [May, 2017, to January, 2018 (9 months)], the orthopedic surgeon (JF) performed 410 primary TKA procedures. Patients who met the inclusion criteria were identified consecutively, and of the 410 procedures, 10 patients (2.4%) met the inclusion criteria and were enrolled in the trial. All 10 participants received the intervention as intended. No patients declined the intervention. Demographics for both groups are reported in Table 1. The MPT group and the MUA group were similar at baseline for age, sex, BMI, preoperative knee flexion and extension ROM, and T0 knee flexion and extension ROM.

**Table 1:** Demographic and baseline data

	MUA, *N* = 31, mean ± SD	MPT, *N* = 10, mean ± SD	*Between-group difference, mean (95%CI)*
Age (years)	64.7 ± 9.2	64.2 ± 9.0	*0.5 (− 6.5, 7.5)*
Sex (% female)	64.5%	70%	*− 5.5% (− 38.5%, 27.5%)*
Body mass index	30.6 ± 5.0	30.5 ± 6.9	*0.0 (− 5.1, 5.2)*
Preop knee flexion ROM (°)	127.0 ± 7.1	129.5 ± 8.3	*− 2.5 (− 8.8, 3.8)*
Preop knee extension ROM (°)	5.3 ± 5.9	3.2 ± 4.6	*2.1 (− 1.7, 5.8)*
T0 knee flexion ROM (°)	86.9 ± 7.4	90.2 ± 5.0	*− 3.3 (− 7.6, 0.9)*
T0 knee extension ROM (°)	4.4 ± 6.1	5.6 ± 6.3	*− 1.2 (− 6.1, 3.6)*
Time from index TKA to T0 (days)	63.0 ± 13.0	50.9 ± 6.3	*12.1 (5.9, 18.4)*

*Abbreviations*: *MUA* manipulation under anesthesia, *MPT* multimodal physical therapy, *ROM* range of motion, *TKA* total knee arthroplasty

### Range of motion

**Fig. 2:**
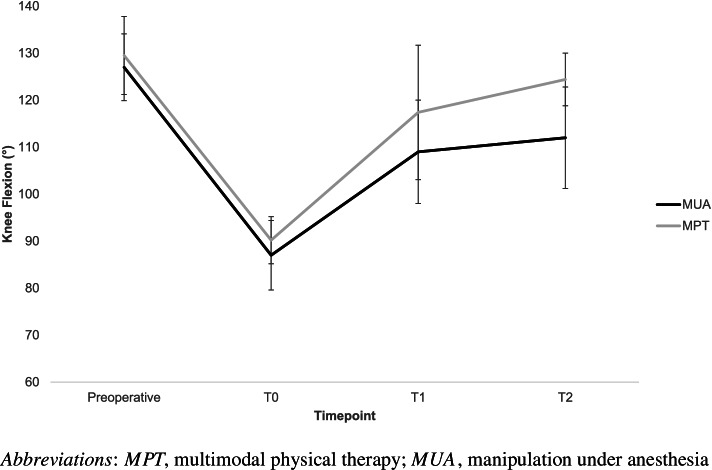
Changes in knee flexion over time

**Fig. 3:**
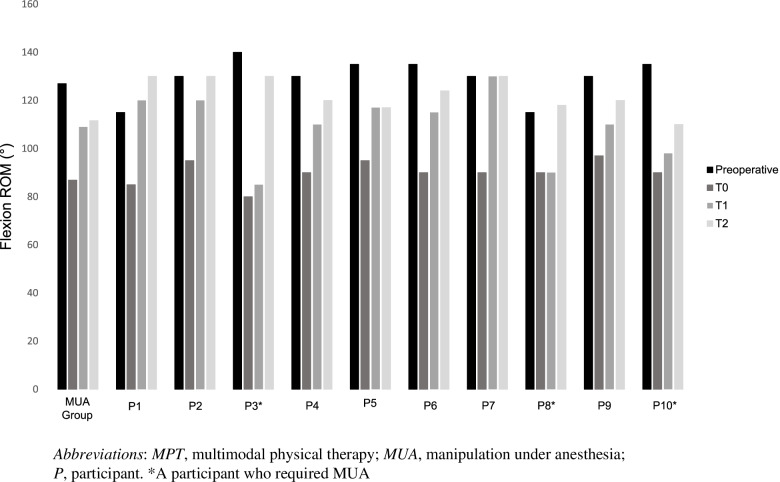
Changes in knee flexion for the MPT group (individual) compared to the MUA group

The range of motion data for both groups is reported in Table 2 and Figs. 2 and 3. At T1 knee flexion, ROM in MPT was similar to MUA with a mean difference of 0.5° (95%CI − 11.2°, 10.2°). Knee extension ROM was also similar between the groups with a mean difference of 3.3° (95%CI − 0.1°, 6.7°). Three individuals in MPT had knee extension deficits of > 5° (mean 13.3 ± 5.7) and utilized the splint for both flexion and extension. All three of these participants achieved the goal of ≥ 110° of knee flexion by T1 and improved their knee extension deficit to 5.7° ± 5.1°.

**Table 2:** ROM differences between the groups

	T1, mean ± SD		*Difference at T1 (MPT-MUA), mean (95%CI)*	T2, mean ± SD		*Difference at T2 (MPT-MUA), mean (95%CI)*
	MUA, *n* = 31	MPT, *n* = 10		MUA, *n* = 31	MPT, *n* = 7	
Knee flexion ROM (°)	109.0 ± 11.0	109.5 ± 14.3	*0.5 (− 10.2, 11.2)*	111.6 ± 10.8	124.4 ± 5.6	*12.8 (4.2, 21.4)*
Knee extension ROM (°)	6.1 ± 7.0	2.8 ± 3.6	*− 3.3 (− 6.7, 0.1)*	4.6 ± 5.2	3.6 ± 5.7	*− 1.0 (− 5.5, 3.5)*

*Abbreviations*: *MUA* manipulation under anesthesia, *MPT* multimodal physical therapy, *ROM* range of motion

Three participants did not improve with the MPT intervention (knee flexion ROM at T1 91.0° ± 6.6°) and subsequently had a MUA. The median (IQR) time from T1 follow-up to MUA was 25 (12–36) days, and time from index TKA was 103 (91–112) days or 14.7 (13.0–16.0) weeks. Two of these participants had a history of prior MUA on their contralateral knee following TKA, and one participant had limited preoperative knee flexion ROM (115°) but no history of prior contralateral TKA. Excluding the participants who required MUA (*n* = 3), the mean ROM at T1 for MPT (*n* = 7) was 117.4° ± 6.9°.

At T2, knee flexion ROM in the MPT (*n* = 7) group was 12.8° (95%CI 21.4°, 4.2°) greater than the MUA group. Knee extension ROM was similar with a mean difference of 1.0° (95%CI − 5.5°, 3.5°) between the groups. At T2, the three participants in MPT who underwent MUA had a knee flexion ROM of 119.2° ± 10.1° resulting in a mean flexion ROM gain following MUA of 28.3° (95%CI 69.3°, − 12.7°).

### Feasibility

Subjects attended all physical therapy visits. Adherence to splint use and satisfaction with splint reached a priori acceptable levels. The mean adherence was 86.6 ± 9.0%. Barriers to adherence were noted to be non-English speaking participants (*n* = 1) due to instructions being available only in English, difficulty sitting for 30 min due to concomitant conditions such as low back pain, and total time to complete the protocol for home splint usage particularly in the combined flexion and extension group (3 h per day total). The median satisfaction was 6 “very satisfied” (interquartile range 5, 7).

One adverse event related to splint use occurred during the study. One participant reported bruising due to the strapping being applied too tightly. This resolved with strapping modification, and no additional treatment was required.

## Discussion

The purpose of this study was to determine the treatment effect, including variability, and feasibility of a MPT program including manual therapy, therapeutic exercise, and static progressive splinting in individuals presenting with early-stage arthrofibrosis (6 weeks postoperative) compared to individuals who underwent MUA. The MPT program was feasible and led to successful outcomes in 70% of participants. Participants attended all physical therapy sessions and rated their satisfaction and adherence with the splint protocol above a priori levels defined for acceptability. There were no significant adverse events related to the MPT program, which included the splint. Our results are consistent with prior literature reporting no adverse events related to splint use [22, 30].

The seven individuals who responded to the MPT program experienced a mean flexion gain of 27.2° at 4 weeks (T1) for a mean flexion ROM of 117.4°. These changes were higher compared to prior literature examining the static progressive splinting with or without additional interventions. One study showed a median knee flexion ROM of 110° (range 64 to 137°) following a median of 7 weeks (range 3 to 16 weeks) of splint use [22]. A second study resulted in mean knee flexion ROM of 107.9° ± 16.8° within a mean of 9.4 ± 7.8 weeks of splint use [24]. These two studies and the current study used the same dosing protocol for the static progressive splint; however, there were key methodological differences between the prior studies and the current study which may account for the differences seen in the results. Bonutti et al. [22] examined the use of a splint after the individuals had already failed to respond to MUA, and the participants did not receive additional physical therapy interventions. Seyler et al. [24] used a decision-making algorithm and applied static progressive splints only for individuals presenting with arthrofibrosis greater than 3 months postoperatively and who had failed to respond to conservative therapy and MUA in some cases. The participants were also eligible to receive other interventions concurrently including continued physical therapy, botulinum toxin injections, and/or electrical stimulation. The current trial used a standardized MPT program which began early in the postoperative phase (6 weeks) and used structured and personalized manual therapy and exercise. These preliminary results suggest the use of the MPT program early in the postoperative phase may be an appropriate alternative to MUA for some patients.

The MPT group demonstrated higher levels of knee flexion ROM at T2 compared to the MUA group by an average of 12.8° which is considered clinically meaningful [31]. Given MUA reinitiates an acute inflammatory process, it may impact ultimate ROM recovery, and future research should evaluate the impact of both MPT and MUA on long-term ROM recovery. It is worth noting that there was a high variability seen in the timing of the final follow-up in both groups (12 months in MPT and 6 months in MUA) which may have influenced the outcomes in both groups. However, prior research indicates there is typically little to no change in ROM between 6 and 12 months after TKA [32], indicating that T2 outcomes observed in this study are likely representative of long-term outcomes in both groups.

The MUA group demonstrated ROM outcomes similar to those reported in prior literature. The mean flexion gain in the MUA group was 24.7° (95%CI 20.8°, 28.7°) which was slightly lower than prior literature which reports a range of 26.1 to 48.9° when MUA is performed within 12 weeks of index TKA [5, 13, 14, 16, 33–36]. However, the final mean flexion ROM of 111.6° (95%CI 107.6°, 115.6°) is towards the higher end of the range cited in prior literature (94.5 to 114°) for the final mean knee flexion when MUA is performed within 12 weeks of index TKA [5, 13, 14, 16, 33–36]. A likely explanation for the differences between the MUA group and prior literature is due to the inclusion criteria necessitating that individuals had preoperative ROM greater than or equal to 110°. Prior studies included subjects with a mean preoperative ROM ranging from 101° to 104.9° [13, 14, 33, 36].

The MPT group achieved similar changes in knee flexion ROM when compared to the MUA group despite having some individuals who did not respond to the intervention (*n* = 3). There are a multitude of factors which may cause arthrofibrosis following TKA [7, 10], and as a result, there is a need to develop care pathways for arthrofibrosis related to these factors to improve outcomes in this population. Using the current theory about the mechanisms and subgroups of arthrofibrosis would help determine who may benefit from early MPT interventions, who may benefit from early MUA, and who may benefit from alternative approaches (e.g., arthroscopy, revision). The results from this feasibility trial suggest one key predictor for positive response may be the presence of higher preoperative ROM (≥ 110°); preoperative ROM is highly predictive of postoperative ROM following TKA [37]. Drawing from current theories [7, 10, 38], the development of early-stage arthrofibrosis may be related to inappropriate management immediately following TKA which could have included inadequate pain control, fear of movement, underdosage of the frequency of ROM exercises, and/or overdosage of the intensity of ROM exercises and manual therapy techniques. The MPT program incorporates frequent knee ROM as part of the home exercise program, requiring individuals to move their knee through non-pain increasing ranges at least five times per day and up to every hour. Overly intense ROM and manual therapy techniques may contribute to arthrofibrosis by increasing pain and inflammation resulting in decreased willingness to move the knee. Historically, manual therapy was explained by biomechanical theories in which it was viewed as a way to “break up” scar tissue by applying forces to structures in order to change the length or mobility of connective tissues [39]; however, a more recent theory about the underlying mechanisms of manual therapy highlights the complexity of the intervention and the interplay among the patient, the provider, and the environment [38]. Drawing from this current theory, it is more likely manual therapy helps improve ROM through neurophysiological mechanisms which aid in the modulation of pain. The manual therapy component of the MPT program was applied using this current theory and was personalized based on the patient’s response. Further research is needed to determine how each part of the MPT program contributes to recovery from early-stage arthrofibrosis.

As seen with this study, the MPT program was not beneficial for all patients presenting with early-stage arthrofibrosis following TKA. Among the individuals who did not respond to the MPT program, two of them had a history of MUA on the contralateral knee following TKA. This may be an indicator of an underlying genetic predisposition increasing the risk of arthrofibrosis. Identification of genetic markers for arthrofibrosis would improve counseling prior to surgery and impact selection of appropriate interventions when arthrofibrosis manifests following TKA. For example, Usher et al. [7] characterizes two distinct phases of arthrofibrosis and posits that early, active phase arthrofibrosis may not respond favorably to MUA because of the tissue disruption and ensuing inflammation which perpetuates fibrosis. However, these individuals may respond more favorably to late MUA once they have transitioned into the stable phase of arthrofibrosis but before scar maturation occurs. This may be an explanation for the variability in the literature examining the timing of MUA with some studies showing superiority in favor of early MUA and some showing no difference in the outcomes based on early versus late timing [11–16]. More research is needed to better identify individuals who would benefit the most from early MUA or the MPT program.

The results of this feasibility study support conducting a larger randomized controlled trial to evaluate the differences between MPT and MUA; however, we anticipate several challenges in conducting a larger study. Incidence of knee fibrosis following TKA is variable and relatively rare with rates of 1.3 to 13% reported in the literature [2–6]. This reduces potential participants by 87 to 98% of all individuals receiving TKA. For the current feasibility trial, it required 9 months to recruit 10 consecutive participants from one orthopedic surgeon; these 10 patients represented 2.4% of all TKA procedures performed in that time frame. Furthermore, it may be difficult to recruit both individuals and surgeons with true equipoise regarding MUA and conservative interventions. Many individuals have strong opinions about which option should be used first, and as a result, it may be difficult to recruit participants willing to be randomized to receive either MUA or four additional weeks of conservative care. This was evident in the METEOR trial which compared arthroscopic meniscectomy to physical therapy for the management of symptomatic meniscal tears in adults aged 45 and older. Researchers were only able to enroll 26.4% of eligible participants, and the primary reason for declining to enroll was a lack of equipoise seen by participants having a strong preference for surgery (36.1%) or physical therapy (21%) [40]. From a surgical perspective, there is disagreement about the appropriate timing of MUA following index TKA as well as how it should be performed. A subsequent multi-site/multi-surgeon trial would need to standardize the timing of postoperative identification and MUA procedure, peri-operative procedures both for index TKA and MUA, and postoperative procedures including pain control and physical therapy protocols (intervention and control).

Subsequent trials should also evaluate the costs associated with both MPT and MUA and subsequent health care utilization. In the current study, the average cost for a monthly JAS splint rental in 2018 was $151 utilizing the average Medicare reimbursed amounts in Colorado. For MUA, the average Medicare reimbursed costs including surgeon fees, hospital fees, and anesthesia fees was $1088. The costs associated with physical therapy were not available for the MPT and MUA groups but may differ depending on the utilization of physical therapy following MUA. Utilization of MPT prior to MUA may also affect the response to MUA although in this study, the three individuals requiring MUA had a satisfactory outcome and similar response to the MUA group.

### Limitations

There are multiple limitations associated with this study. Due to the nature of a feasibility study, the sample size was small (*n* = 10), and it was not powered to detect the differences between the MPT and MUA groups. There was also only one orthopedic surgeon (JF) who performed both the TKA and MUA procedures; as a result, the outcomes may be less generalizable to other surgeons and surgical techniques. There was also one difference at baseline between the MPT and MUA groups; the time from index TKA to initiation of MPT program was shorter by 12 days on average than the time from index TKA to MUA. It is unclear if this difference in timing of the interventions had an impact on outcomes. This 12-day difference is to be expected, however, based on the protocol used to time the MUA. Patients were routinely seen in the clinic 6 weeks postoperatively. If there was a concern for arthrofibrosis, they were given specific instructions to increase their efforts at PT and were followed up 2 weeks later. If patients demonstrated a lack of progression during that additional 2 weeks, they were booked for a MUA within the next few days. On the other hand, patients seen at 6-week follow-up who were randomized to the MPT group began the MPT program immediately (within a few days). Furthermore, it is unknown if patients in either group may have improved without intervention as the natural history of arthrofibrosis is poorly defined as it is often intervened upon (conservatively or surgically). Finally, physical therapy was not standardized in the MUA group which may have impacted the outcomes for this group. The protocol was standardized in the MPT group, all visits were attended, and physical therapists received training and monitoring throughout the protocol. However, we did not use a standardized fidelity plan for all aspects of the intervention, and future efficacy studies should include a structured plan for fidelity to maximize validity.

## Conclusions

The treatment of arthrofibrosis following TKA is variable and can result in poor outcomes and adverse events. Consequently, there is a need for alternative interventions to address this population. This feasibility study showed the MPT program resulted in similar results compared to MUA and was acceptable and satisfactory to participants. Further pilot work may be necessary to test recruitment strategies and alternative study designs (e.g., sequential multiple assignment randomized trial (SMART) design [41], randomized cluster design [42]) which may help overcome the challenges of studying this research question (e.g., lack of equipoise, rarity of arthrofibrosis, standardizing surgical and non-surgical interventions). The results from this study will also help inform a larger randomized controlled trial comparing the MPT program to MUA. Such a study could test the efficacy of the MPT program in addition to improving the identification of individuals who will be more likely to respond to the MPT intervention or MUA for treatment of early-stage arthrofibrosis.

## Supplementary Information

**Additional file 1.** Multimodal Physical Therapy Program.

**Additional file 2.** Supplemental TIDier (Template for Intervention Description and Replication) Information.

## Data Availability

The datasets used and/or analyzed during the current study are available from the corresponding author on reasonable request.

## Abbreviations

MPT: Multimodal physical therapy
MUA: Manipulation under anesthesia
OA: Osteoarthritis
ROM: Range of motion
TKA: Total knee arthroplasty
