# Supplementary material for: Multimodal conservative management of arthrofibrosis after total knee arthroplasty compared to manipulation under anesthesia: a feasibility study with retrospective cohort comparison
Source: Pilot Feasibility Stud. 2022 Mar 25;8:71. doi: 10.1186/s40814-022-01026-y (PMC8953056; doi:10.1186/s40814-022-01026-y)
Supplement: Supplementary file 1 — Additional file 1. Multimodal Physical Therapy Program. [file 40814_2022_1026_MOESM1_ESM.docx]

**Additional file 1. Multimodal Physical Therapy Program**

**Overview**

- The major goal of this protocol is to improve functional mobility by focusing on improved soft tissue/joint mobility and pain modulation. Tolerance criteria (see below) are utilized to assess response to the program in addition to range of motion (ROM) progress.
- The primary focus of this study is on improving knee flexion however many patients have limitations in both flexion and extension. Patients with extension limitations >5° should also work on extension in addition to flexion with the static progressive splint as well as add the multi-modal extension deficit protocol to their treatment plan (see below). Patients will continue brace use until they are able to extend to neutral.
  - Patients with and extension deficit of less than 5° should be encouraged to stretch 30 min daily until full extension is achieved. In addition, if a patient presents with less than full extension add the multi-modal extension deficit protocol to their treatment plan until neutral extension is possible

**Multimodal Physical Therapy Program Components**

*Manual Therapy*

- There are core manual therapy techniques and optional manual therapy techniques. Core techniques should be utilized at the initial evaluation and a test-retest approach should be utilized to determine patient response. Utilization of optional techniques is based upon patient presentation, clinical reasoning, and a test-retest approach.[1]
- Mandatory techniques should be utilized during the initial treatment sessions to determine patient’s responsiveness. Techniques that are determined to be ineffective for the patient should be discontinued. A test-retest approach is encouraged for all techniques.
- An active-assisted ROM approach (including manual resistance) may be considered for individuals who do not respond favorably to manual therapy techniques (typically present with a empty/guarded end feel during passive ROM)
- For individuals who present with a boggy (swollen) end feel during passive ROM consider modifying position of manual therapy techniques by elevating the patient’s limbs and utilizing an elevated position for warm-up. Mid-range techniques will also be prioritized in this situation to promote normalization of edema. In addition, techniques to reduce edema (compression, elevation, active muscular contraction) will be prioritized initially.
- Flexion deficit protocol (all patients)
  - Mandatory Techniques (based on type of implant)
    - Soft-tissue mobilization to quadriceps, adductors, surgical incision, peri-patellar tissues
      - Also assigned as part of the home exercise program (HEP) to be performed daily
    - Patellofemoral mobilization
      - General rotational at limit of extension
      - Specific assessment of restriction in a clockwise fashion
      - Work in extended position first and increase knee flexion as able
      - Also assigned as part of HEP to be performed daily
    - Tibiofemoral ROM with distraction
      - Begin with general mid-range technique first with progression into restricted range
    - Tibiofemoral anterior to posterior and posterior to anterior mobilization in seated at limit of flexion
      - Rotation added depending on implant
    - Finish with active ROM with manual resistance through full range
  - Optional Techniques
    - Tibiofemoral distraction and varus or valgus stress (popliteal wedge optional)
    - Tibiofemoral mobilization with movement (MWM) medial or lateral glides in non-weight bearing or weight bearing [2]
    - Patellofemoral MWM
    - Potential use of contract relax techniques to decrease muscle guarding
    - Potential use of soft tissue mobilization during ROM to decrease pain

- - Extension deficit protocol (If extension >0 (flexion contracture))
    - Mandatory Techniques
      - Soft-tissue mobilization to the hamstrings and gastroc/soleus
        - Ensure there is no muscle guarding when patient is positioned in extension
        - Also assigned as part of HEP to be performed daily
      - Tibiofemoral ROM with distraction
        - Begin with general mid-range technique first with progression into end-range
        - Varus and valgus added to increase comfort and progress range
      - Tibiofemoral anterior to posterior and posterior to anterior mobilization in extension with distraction
      - Finish with active ROM with manual resistance through full range
    - Optional Techniques
      - Tibiofemoral MWM medial or lateral glides in non-weight bearing or weight bearing
      - Potential use of contract relax to decrease muscle guarding
      - Potential use of soft tissue mobilization during ROM to decrease pain
  - Additional optional techniques targeting the lumbar spine, hips, ankle and foot could be added based upon patient presentation, clinical reasoning, and a test-retest approach

*Therapeutic Exercise*

- Active-assisted ROM, active ROM, cycling, or recumbent stepper (patient choice) with focus on non-pain increasing ROM. These should be pain relieving and very comfortable. The goal with these exercises is to teach that movement is not painful, provide increased circulation to the joint, and prepare the tissues for deeper stretching. Typically, these exercises can be utilized as a warm-up to clinic sessions and to decrease post-splint/manual therapy/exercise feelings of tightness that can occur from prolonged positioning or bouts of more intense exercise where co-contraction is a common occurrence.
- Active-assisted ROM and active ROM exercises are dosed in the home exercise program (HEP) as 10 repetitions hourly while the patient is awake. By providing multiple methods of incorporating ROM adherence is improved.
  - Preferred AAROM exercises are: step-stretch, heel slides with a belt, sheet or towel for assistance, or seated knee extension with a belt, sheet or towel.
  - Preferred AROM exercises are: seated leg-extensions, heel slides, recumbent stepper, and cycling. Use of the recumbent stepper and cycling is only added once the patient is proficient in all other techniques so they will be able to utilize these at home as part of their HEP. Cycling is recommended for individuals with knee flexion >100° and if they enjoy cycling. No loads are applied with cycling until full flexion ROM is achieved. Use of loads during use of the recumbent stepper or cycling will be dosed at an RPE of 4 (somewhat hard) on a 0-10 rating of perceived exertion (RPE) scale after ROM is progressed.
- Flexibility exercises are instructed as part of HEP and completed in clinic if time and based upon traditional assessments of muscle length for inclusion into the HEP. These are dosed at three times daily for a minimum of one-minute holds for each repetition. Contract relax techniques may be employed to facilitate relaxation and minimize muscle guarding if present. Common muscles targeted are:
  - Quadriceps (flexion deficit)
  - Hamstrings and Gastrocnemius (extension deficit)
- Task Training is utilized to encourage the utilization of flexion and extension ROM gained during the activities of standing, walking, rising from a chair, sitting, kneeling and stair climbing. Participants are encouraged to intentionally perform these tasks several times daily to facilitate incorporation into habitual movements.
- Additional exercises targeting deficits in strength and balance may be prescribed based upon patient presentation.

*Static Progressive Splinting*

- A static progressive splint (Joint Active Systems SPS Knee, Effingham, IL) is custom fit to the patient
- Gradually increase use the splint to three times per day, 30-minutes a session, for a total of 90 minutes per day for each direction utilized
- Instruct to increase the stretch delivered by the splint to a level of 2-3 (light stretch) out of 10 where 0 equaled “no stretch” and 10 equaled “painful stretch”
- Every five minutes, patients were instructed to evaluate the level of stretch and increase or decrease the splint tension to maintain a level of 2-3 throughout the entire session

*Education*

- General Education:
  - Troubleshooting problems with HEP and usage of the JAS Splint
  - Education on typical recovery time frames and establishing appropriate expectations
  - Swelling management strategies (if appropriate)
- Pain Management:
  - Proper dosing of pain medications. Do not take medications purposefully prior to therapy as patient perceptions are dulled and over-stressing is more likely. Encourage staying within a consistent cycle of use with gradual weening.
  - Movement is healthy. Strategies to increase pain-free movement throughout the day are key.
  - Use of pedometer (smart phone) to try and maintain consistent level of activity as large variations in daily activity can lead to fluxes in pain and swelling levels.
  - Use of modalities (ice/heat) to facilitate improved mobility and pain reduction
- Tolerance criteria:
  - - Decrease in ROM by 5° from last treatment
    - Increase of more than 2 points in resting pain
    - Soreness lasting for greater than 2 hours
    - Subjective decrease in ability to sit to stand or walk short distances
  - If patient has one of the findings above, maintain current level of provocative exercise/s and advance all others as tolerated
  - If the patient has two or more findings above decrease treatment intensity to a previous level and focus on ROM/low-intensity/low-load exercise for that session

Stopping Criteria

If patients have not achieved 110° by the 10-week postoperative time point (4 weeks of total splint use) or demonstrated a 10° improvement in flexion ROM over the first 4 weeks of splint use, then the utilization of manipulation under anesthesia will be discussed with the patient. Patients will decide at this time to either discontinue use of the splint in favor of surgical management (MUA) or continue with splint use. Should patients wish to continue with splint use at this time as they have not yet achieved 110° but have continued to demonstrate progress a research assistant will measure knee ROM every two weeks. Knee ROM measurements will occur via a home visit. Splint use will be discontinued once 110° of knee flexion is achieved or there is a two-week plateau in knee ROM gains. Patients will be able to undergo MUA at any time after the 10-week postoperative time point should they elect to.

References

1. Hengeveld E, Banks K. Maitland's Vertebral Manipulation, Volume 1, 8e and Mailand's Peripheral Manipulation, Volume 2, 5e: Managment of Musculoskeletal Disorders - Volumes 1 & 2: Churchill Livingstone, 2014

2. Vicenzino B, Hing W, Rivett D, Hall T. Mobilisation with Movement: The Art and the Science: Churchill Livingstone Australia, 2011
