# Supplementary material for: Multimodal conservative management of arthrofibrosis after total knee arthroplasty compared to manipulation under anesthesia: a feasibility study with retrospective cohort comparison
Source: Pilot Feasibility Stud. 2022 Mar 25;8:71. doi: 10.1186/s40814-022-01026-y (PMC8953056; doi:10.1186/s40814-022-01026-y)
Supplement: Supplementary file 2 — Additional file 2. Supplemental TIDier (Template for Intervention Description and Replication) Information. [file 40814_2022_1026_MOESM2_ESM.docx]

**Additional File 2. Supplemental TIDier (Template for Intervention Description and Replication) Information**

| Item | Details |
| --- | --- |
| BRIEF NAME: Provide the name or a phrase that describes the intervention | Multimodal physical therapy program |
| WHY: Describe any rationale, theory, or goal of the elements essential to the intervention. | Drawing from current theories the development of early stage arthrofibrosis may be related to inappropriate management immediately following TKA which could have included inadequate pain control, fear of movement, underdosage of the frequency of ROM exercises, and/or overdosage of the intensity of ROM exercises and manual therapy techniques. The MPT program incorporates frequent knee ROM as part of the home exercise program, requiring individuals to move their knee through non-pain increasing ranges at least five times per day and up to every hour. Overly intense ROM and manual therapy techniques may contribute to arthrofibrosis by increasing pain and inflammation resulting in decreased willingness to move the knee. Historically, manual therapy was explained by biomechanical theories in which it was viewed as a way to “break up” scar tissue by applying forces to structures in order to change the length or mobility of connective tissues; however, a more recent theory about the underlying mechanisms of manual therapy highlights the complexity of the intervention, and the interplay among the patient, the provider, and the environment. Drawing from this current theory, it is more likely manual therapy helps improve ROM through neurophysiological mechanisms which aid in the modulation of pain. The manual therapy component of the MPT program was applied using this current theory and was personalized based on the patient’s response. The static progressive splint utilized in the intervention is theorized to remodel tissue utilizing a stress hysteresis theory where a low-load, long-duration force causes tissue creep in response to the prolonged force allowing for increased joint mobility. |
| WHAT: Materials: Describe any physical or informational materials used in the intervention, including those provided to participants or used in the intervention delivery or training of intervention providers. | Therapists received a handout of Additional File 1 for utilization in training. Participants received an adherence log, an instruction manual for the static progressive splint, and pictures/instructions for their home exercises. |
| WHAT: Procedures: Describe each of the procedures, activities, and/or processes used in the intervention, including any enabling or support activities. | Core manual therapy techniques (See Additional File 1) were utilized at the initial evaluation to determine patient response and optimal technique selection. Techniques that facilitated improved within-session gains of ROM and/or reductions of pain were utilized throughout the MPT program. Manual therapy techniques that were easy to apply by the patient (e.g., patellofemoral mobilizations or soft tissue mobilization) were also prescribed as part of a home exercise program to be performed daily as appropriate. All patients were prescribed an active-assisted ROM exercise to be performed at least five times per day (recommended hourly while awake) and instructed to complete this exercise to their current limit of flexion and extension in a non-pain increasing manner. Patients were also prescribed flexibility exercises to be performed three times daily for 60-seconds each. Finally, patients were prescribed weight-bearing and task specific exercises to be performed daily to incorporate ROM gains and facilitate strength gains within newly acquired ranges. Exercises were determined by the treating therapist and not standardized.  A static progressive splint (Joint Active Systems SPS Knee, Effingham, IL) was custom fit to the patient. They were instructed to gradually increase use the splint to three times per day, 30-minutes a session, for a total of 90 minutes per day for each direction utilized. They were instructed to increase the stretch delivered by the splint to a level of 2-3 (light stretch) out of 10 where 0 equaled “no stretch” and 10 equaled “painful stretch”. Every five minutes, patients were instructed to evaluate the level of stretch and increase or decrease the splint tension to maintain a level of 2-3 throughout the entire session |
| HOW: Describe the modes of delivery of the intervention and whether it was provided individually or in a group. | In-person delivery of the intervention provided individually. |
| WHERE: Describe the type(s) of location(s) where the intervention occurred, including any necessary infrastructure or relevant features. | Two outpatient physical therapy clinics located in the Denver metro area. |
| WHEN and HOW MUCH: Describe the number of times the intervention was delivered and over what period of time including the number of sessions, their schedule, and their duration, intensity or dose. | Participants were seen twice per week for 4 weeks for a total of 8 visits. Average session duration was 1 hour. |
| TAILORING: If the intervention was planned to be personalized, titrated or adapted, then describe what, why, when, and how. | There are core manual therapy techniques and optional manual therapy techniques. Core techniques should be utilized at the initial evaluation and a test-retest approach should be utilized to determine patient response. Utilization of optional techniques is based upon patient presentation, clinical reasoning, and a test-retest approach. Mandatory techniques should be utilized during the initial treatment sessions to determine patient’s responsiveness. Techniques that are determined to be ineffective for the patient should be discontinued. A test-retest approach is encouraged for all techniques. A positive response to a technique was determine if pain with end-range motion decreased and/or range of motion increased following utilization.  Tolerance criteria utilized for therapeutic exercises:   - Decrease in ROM by 5° from last treatment - Increase of more than 2 points in resting pain - Soreness lasting for greater than 2 hours - Subjective decrease in ability to sit to stand or walk short distances   If patient has one of the findings above, maintain current level of provocative exercise/s and advance all others as tolerated. If the patient has two or more findings above decrease treatment intensity to a previous level and focus on ROM/low-intensity/low-load exercise for that session |
| MODIFICATIONS: If the intervention was modified during the course of the study, describe the changes. | Not applicable |
| HOW WELL: Planned: If the intervention adherence or fidelity was assessed, describe how and by whom, and if any strategies were used to maintain or improve fidelity, describe them. | Intervention adherence was assessed using adherence logs completed by the participants daily. Fidelity of the intervention was assessed by direct observation of patient treatment sessions twice over the four week period for each participant. Observations were conducted by a member of the investigative team (MB) who is a licensed physical therapist. Therapists were given feedback on each session as well as an opportunity to clarify any aspect of the intervention. |
| HOW WELL: Actual: If intervention adherence or fidelity was assessed, described the extent to which the intervention was delivered as planned. | Mean adherence was 86.6 ± 9.0%. A formal fidelity checklist for intervention adherence was not utilized for this trial. |
